# Emulation of a Target Trial From Observational Data to Compare Effectiveness of Casirivimab/Imdevimab and Bamlanivimab/Etesevimab for Early Treatment of Non-Hospitalized Patients With COVID-19

**DOI:** 10.3389/fimmu.2022.868020

**Published:** 2022-04-20

**Authors:** Valentina Mazzotta, Alessandro Cozzi-Lepri, Francesca Colavita, Simone Lanini, Silvia Rosati, Eleonora Lalle, Ilaria Mastrorosa, Claudia Cimaglia, Alessandra Vergori, Nazario Bevilacqua, Daniele Lapa, Andrea Mariano, Aurora Bettini, Chiara Agrati, Pierluca Piselli, Enrico Girardi, Concetta Castilletti, Anna Rosa Garbuglia, Francesco Vaia, Emanuele Nicastri, Andrea Antinori

**Affiliations:** ^1^ Clinical and Infectious Diseases Research Department, National Institute for Infectious Diseases Lazzaro Spallanzani Istituiti di Ricovero e Cura a Carattere Scientifico (IRCCS), Roma, Italy; ^2^ Centre for Clinical Research, Epidemiology, Modelling and Evaluation (CREME), Institute for Global Health, University College London (UCL), London, United Kingdom; ^3^ Laboratory of Virology, National Institute for Infectious Diseases Lazzaro Spallanzani Istituiti di Ricovero e Cura a Carattere Scientifico (IRCCS), Roma, Italy; ^4^ Clinical Epidemiology, National Institute for Infectious Diseases Lazzaro Spallanzani Istituiti di Ricovero e Cura a Carattere Scientifico (IRCCS), Roma, Italy; ^5^ Laboratory of Cellular Immunology and Pharmacology, National Institute for Infectious Diseases Lazzaro Spallanzani Istituiti di Ricovero e Cura a Carattere Scientifico (IRCCS), Roma, Italy; ^6^ Scientific Direction, National Institute for Infectious Diseases Lazzaro Spallanzani Istituiti di Ricovero e Cura a Carattere Scientifico (IRCCS), Roma, Italy; ^7^ Health Direction, National Institute for Infectious Diseases Lazzaro Spallanzani Istituiti di Ricovero e Cura a Carattere Scientifico (IRCCS), Roma, Italy

**Keywords:** monoclonal antibodies, SARS-COV-2, COVID-19, casirivimab/imdevimab, bamlanivimab/etesevimab, early treatment for COVID-19

## Abstract

**Objectives:**

Comparative analysis between different monoclonal antibodies (mAbs) against SARS-CoV-2 are lacking. We present an emulation trial from observational data to compare effectiveness of Bamlanivimab/Etesevimab (BAM/ETE) and Casirivimab/Imdevimab (CAS/IMD) in outpatients with early mild-to-moderate COVID-19 in a real-world scenario of variants of concern (VoCs) from Alpha to Delta.

**Methods:**

Allocation to treatment was subject to mAbs availability, and the measured factors were not used to determine which combination to use. Patients were followed through day 30. Viral load was measured by cycle threshold (CT) on D1 (baseline) and D7.

Primary outcome was time to COVID-19-related hospitalization or death from any cause over days 0-30. Weighted pooled logistic regression and marginal structural Cox model by inverse probability weights were used to compare BAM/ETE vs. CAS/IMD. ANCOVA was used to compare mean D7 CT values by intervention. Models were adjusted for calendar month, MASS score and VoCs. We evaluated effect measure modification by VoCs, vaccination, D1 CT levels and enrolment period.

**Results:**

COVID19-related hospitalization or death from any cause occurred in 15 of 237 patients in the BAM/ETE group (6.3%) and in 4 of 196 patients in the CAS/IMD group (2.0%) (relative risk reduction [1 minus the relative risk] 72%; p=0.024). Subset analysis carried no evidence that the effect of the intervention was different across stratification factors. There was no evidence in viral load reduction from baseline through day 7 across the two groups (+0.17, 95% -1.41;+1.74, p=0.83). Among patients who experienced primary outcome, none showed a negative RT-PCR test in nasopharyngeal swab (p=0.009) and 82.4% showed still high viral load (p<0.001) on D7.

**Conclusions:**

In a pre-Omicron epidemiologic scenario, CAS/IMD reduced risk of clinical progression of COVID-19 compared to BAM/ETE. This effect was not associated with a concomitant difference in virological response.

## Introduction

The widespread of severe acute respiratory syndrome coronavirus 2 (SARS-CoV-2) infection, causing coronavirus disease-2019 (COVID-19), continues to be a challenge for global public health.

Monoclonal antibodies (mAbs) against SARS-CoV-2 have emerged as the strategy of choice for the treatment of early mild-to-moderate COVID-19 in outpatients at increased risk of clinical progression (1) and, based on data from randomized clinical trials (RCT), Bamlanivimab/Etesevimab (2, 3), Casirivimab/Imdevimab (4, 5)and Sotrovimab (6) received emergency use authorizations by the Italian Medicines Agency (AIFA) (7).

In addition to RCT, data from observational cohorts (8–11)have also confirmed the effectiveness of mAbs, but comparative analysis between different options available are lacking (12, 13). Considering the rapid epidemiological evolution, with the emergence of new Variants of Concern (VoCs) (14–16) that have been shown to escape (17) the action of mAbs *in vitro* (18, 19) and *in vivo* (20), real-life data about clinical impact and mAb comparison are useful to better clarify the scenario of currently existing drugs.

The aim of this analysis was to compare the clinical effectiveness of two mAb combinations, Bamlanivimab/Etesevimab (BAM/ETE) and Casirivimab/Imdevimab (CAS/IMD), in a real-life setting during a period in which the prevalent lineages in Italy were B.1.1.7 (Alpha) and B.1.617.2 (Delta), and B.1.529 (Omicron) was not yet circulating.

## Methods

### Monoclonal Antibody Access Program and Eligible Patients

On March 2021, mAb administration program started at the National Institute for Infectious Diseases Lazzaro Spallanzani IRCCS in Rome, ruled by AIFA. Eligibility criteria included outpatients with a confirmed diagnosis of SARS-CoV-2 infection by an antigenic or molecular nasopharyngeal swab (NPS), mild or moderate symptoms of COVID-19 for 10 days or less and at least one of following risk factors for progression to severe disease: body mass index (BMI) ≥35, chronical peritoneal dialysis or hemodialysis, uncontrolled or complicated diabetes mellitus, primary or secondary immunodeficiency. Subjects who were 55 years and older were also eligible if they had any cardio-cerebrovascular diseases, Chronic Obstructive Pulmonary Disease (COPD) or other chronic respiratory diseases. Patients requiring hospitalization for COVID-19 or supplemental oxygen therapy were excluded. In June 2021 (21), AIFA expanded use of mAbs including all patients with one of the following: 65 years and older, BMI >30, any chronic renal impairment (including subjects undergoing peritoneal dialysis or hemodialysis), uncontrolled or complicated diabetes mellitus, any immunocompromising condition [including primary and secondary immunodeficiencies (1)], cardio-cerebrovascular diseases (including hypertension with concomitant organ damage), COPD or other chronic respiratory diseases, chronic liver disease, hemoglobinopathies, neurodevelopmental and neurodegenerative disease.

Comorbidity burden was assessed using Monoclonal Antibodies Screening Score [MASS (7–22)] that assigned points, as follows: age ≥65 (2 points), BMI ≥35 (1 point), diabetes mellitus (2 points), chronic kidney disease (3 points), cardiovascular disease in a patient ≥55 years (2 points), chronic respiratory disease in a patient ≥55 years (2 points), hypertension in a patient ≥55 years (1 point), and immunocompromised status (3 points).

All consecutive adult patient (age>18) who provided a written informed consent were included in study population. The study was approved by AIFA and National Ethics Committee.

### Monoclonal Antibody Administration

Bamlanivimab/Etesevimab (700 mg/1400 mg) or Casirivimab/Imdevimab (1200 mg/1200mg) were administered by one-hour intravenous infusion and patients were observed for one hour after infusion. Allocation to treatments was pseudo-random, as a criterion of daily alternation (subject to drugs availability) was adopted, and as not many of the measured factors were used to determine which combination to infuse.

Patients who received Bamlanivimab as monotherapy or Sotrovimab were excluded.

### Procedures and Data Collection

Outpatients visits were scheduled at baseline (D1) and at 7 (D7) and 30 (D30) days after infusion. Medical evaluation, vital signs recording, laboratory tests and reports on adverse effects were performed at each visit. If patients missed person visits, they were called by telephone to assess clinical conditions. As a real-life study, due to the high diagnostic demands related to the COVID-19 pandemic, different methods were used to investigate serology and virological parameters according to the laboratory workflow and tests availability.

SARS-COV-2 serology was performed by ELISA detecting anti-SARS-CoV-2 IgG, IgM, and IgA (ENZY-WELL SARS-CoV-2; DIESSE, Diagnostica Senese, Siena, Italy; positive index values ≥1.1), or by two chemiluminescence microparticle assays (CMIA) detecting anti-Nucleoprotein and anti-Spike/RBD IgG (ARCHITECT SARS-CoV-2 IgG, and ARCHITECT SARS-CoV-2 IgG II Quantitative; Abbott Laboratories, Wiesbaden, Germany, respectively). According to the to manufacturer’s instructions, for the two CMIA, Index >1.4 and Binding Antibody Units (BAU)/mL ≥7.1 are considered positive for anti-N and anti-Spike/RBD IgG, respectively.

Semi-quantitative estimation of viral load in NPS was assessed by RT-PCR using DiaSorin Simplexa^®^ COVID-19 Direct platform (DiaSorin, Saluggia, Italy), based on cycle threshold (CT) values of S and ORF1ab genes amplification. Other RT-PCR methods used to verify the presence of SARS-CoV-2 were the Abbott m2000 RealTime System (Abbott Laboratories, Wiesbaden, Germany) and the Cobas^®^ SARS-CoV-2 Test on the fully-automated cobas^®^ 6800 Systems (Roche Diagnostics, Rotkreuz, Switzerland).

Identification of VoCs was conducted by Sanger sequencing of the Spike coding gene on the D1 samples. During the period of Delta variant wave, the RT-PCR Simplexa^®^ SARS-CoV-2 Variants Direct kit (DiaSorin, Saluggia, Italy). was included in the study as rapid method for the qualitative detection and differentiation of the N501Y, E484K, E484Q and L452R mutations.

### Outcomes

The primary outcome was defined as time to hospitalization due to development of severe COVID-19 or death from any cause over days 0-30. Secondary outcomes were a) time to hospitalization or death from any cause by day 30, b) time to hospitalization or death from any cause over days 3-30, c) the impact of intervention on CT values change from baseline to D7. The proportion of participants reporting adverse events were also shown as a safety endpoint.

### Statistical Analysis

Main characteristics of the participants, assessed at D1, were compared by treatment strategy using Mann-Whitney U test for continuous variables, expressed as median (IQR) or χ^2^ test or Fisher’s exact test as appropriate for Categorical variables expressed as numbers and percentages.

**Figure 1:**
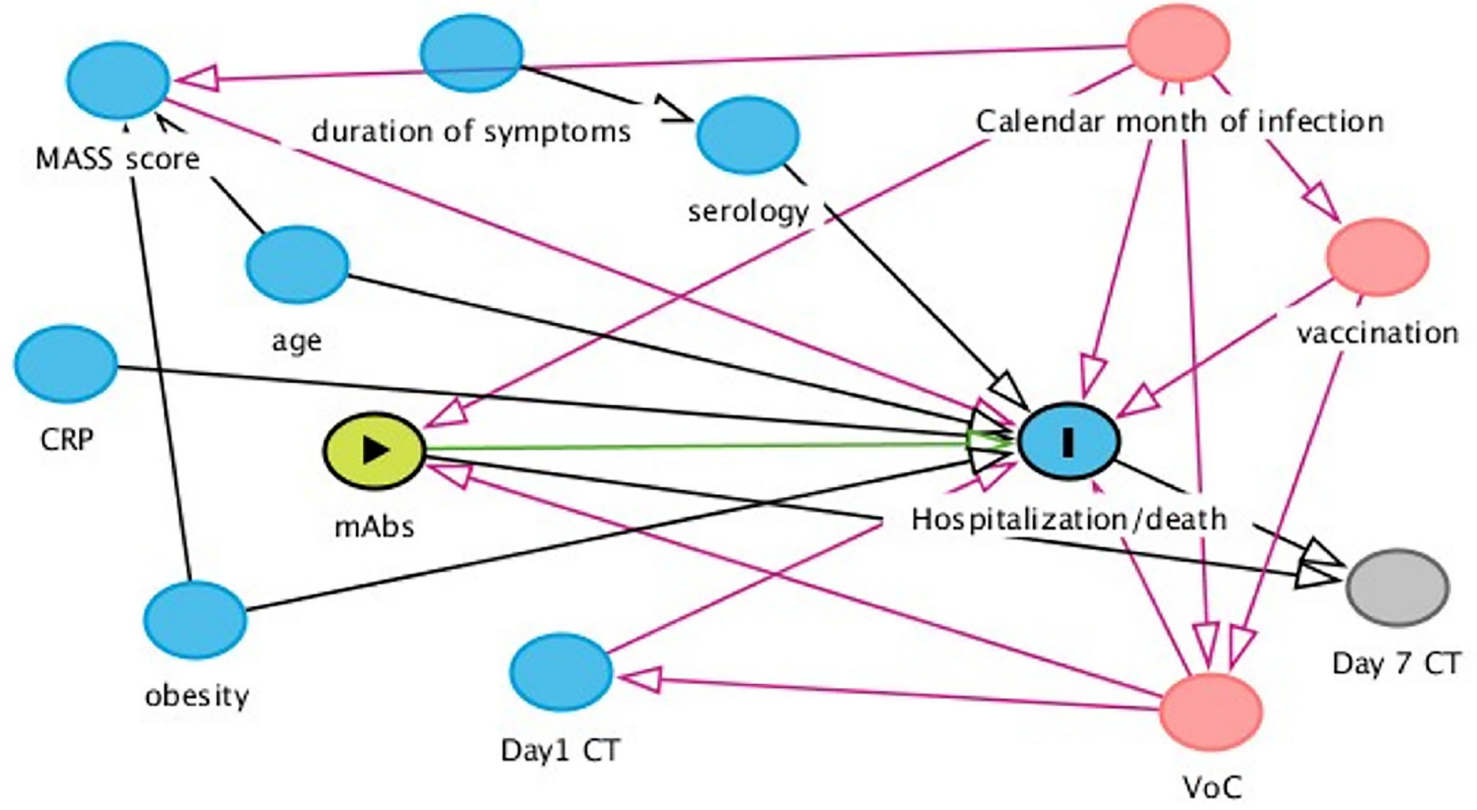
Assumptions regarding the underlying causal link between measured factors. According to our assumptions, month of infusion and type of VoC were identified as main time-fixed confounders of our comparison of interest. MASS score was an important predictor and used in some of the models to increase efficiency. Serology test was performed in all participants on the day of the infusion. As a consequence, the result of the test was not known to the prescriber when the intervention was initiated. Consequently, serology cannot be a confounder for the association of interest.

The effectiveness of the two strategies on the three outcomes was estimated and compared using a weighted pooled logistic regression model which approximates the parameters of a marginal structural Cox model by mean of inverse probability weights. Participants’ follow-up accrued from the date of infusion until the date of hospitalisation, death or date of discharge. Administrative censoring was also applied at 31/12/2021 the date at which the database was frozen. Weights have been calculated using the predicted values from the pooled logistic models for the probabilities of starting BAM/ETE vs. CAS/IMD and those of censoring, respectively. Treatment was fitted as a time-fixed variable and there was no need to account for immortal time bias. Potential informative censoring was controlled for using inverse probability of censoring weights. Unweighted and weighted hazard ratios (HRs) with 95% confidence intervals (CI) were shown. Assumptions regarding the underlying causal link between measured factors are pictured in **Figure 1**. Unweighted and weighting Kaplan-Meier estimates of the primary outcome stratified by treatment strategy were fitted. Interactions between the intervention and study population strata (type of VoC, vaccination status, baseline SARS-COV-2 serology, level of D1 CT values and period of enrolment) were formally tested by including a multiplicative term in the marginal Cox regression model and adjusted hazard ratios (HRs) with 95% confidence intervals (CI) were shown in a forest plot.

Positive serology was defined as detection of IgG and/or IgA with ELISA or detection of IgG anti-S and/or anti-N by CMIA.

We compared mean CT values at D7 by treatment in analysis of covariance (ANCOVA- model adjusted for D1 CT value, month of enrolment and type of VoC) and we described, using box-plots, median (IQR) of D1 and D7 CT values and its variation.

We also evaluated the associations between D7 CT response (using the cut-offs of 40 for negativity and 25 for reduced viral load) with both the intervention and the primary outcome using a chi-square and Fisher exact test as appropriate.

A descriptive analysis of self-reported side effects was also performed.

A two-sided test of less than 0.05 was considered statistically significant. All statistical analyses were performed using the SAS software, version 9.4 (Carey USA).

## Results

### Study Population

**Figure 2:**
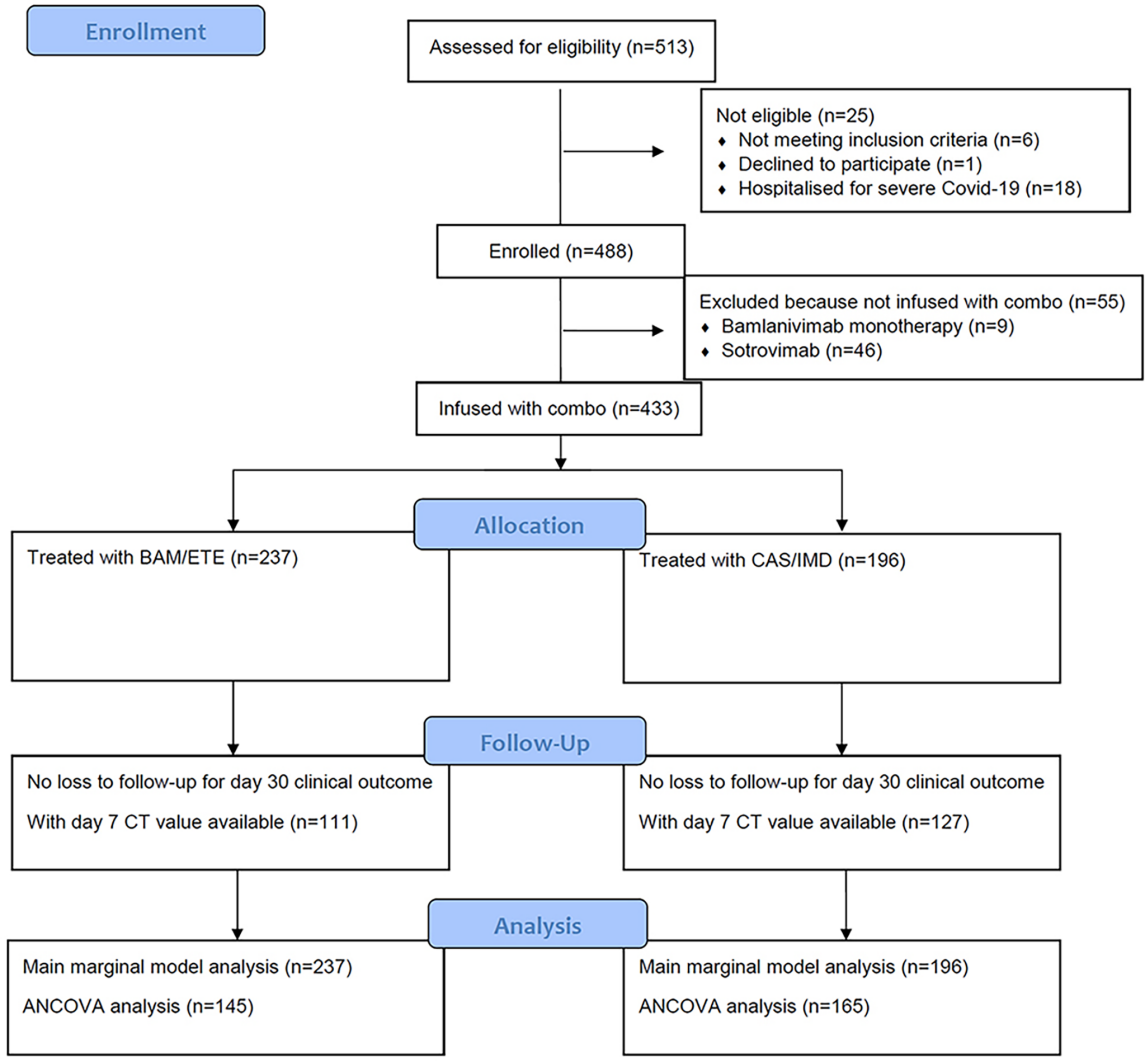
Study flow chart.

From 23^rd^ March 2021 to the 3^rd^ of December 2021, a total of 513 participants were evaluated for receiving mAb treatment and 433 of them (84.4%) were included in the analysis (**Figure 2**). Briefly, 201 (46%) were female, median age was 63 years (IQR 53-73), and 241 patients (57%) were vaccinated. At baseline, median MASS score was 2 (IQR 0-4) and median time from symptoms onset to D1 was 5 days (IQR 3-6). Alfa (B.1.1.7) variant were identified in 71 participants (22%), Gamma (P.1) in 25 (8%) and Delta (B.1.617.2) in 192 (59%); Beta (B.1.135) and Eta (B.1.525) in one participant each. Negative SARS-COV-2 serology at D1 was detected in 154 (35.6%) participants. Serology test results were available for 199 of the 241 vaccinated participants (83%). Of the 61 vaccinated patients tested with ELISA, 41 (67%) showed a positive SARS-COV-2 serology; of the 138 vaccinated patients tested by CMIA, 130 (94%) were positive to anti-Spike antibodies, and only 7 were positive to anti-N antibodies. Among vaccinated patients, mRNA-1273 was used more frequently in patients receiving BAM/ETE, and ChAdOx1 less frequently (p=0.05). Overall, the participants receiving the two treatment strategies appeared to be balanced with respect to key predictors of outcomes as expected under our pseudo-random allocation design. Main characteristics according to the two treatment groups are reported in **Table 1**.

**Table 1:** Main characteristics at enrolment by intervention group.

Characteristics	Regimen started			
	BAM/ETE	CAS/IMD	p-value^*^	Total
	N = 237	N = 196		N = 433
***Gender, n (%)***			0.701	
Female	112 (47.3%)	89 (45.4%)		201 (46.4%)
***Age, years***			0.798	
Median (IQR)	63 (53, 73)	62 (53, 74)		63 (53, 73)
***Days from sypmtoms onset to MAbs infusion***			0.624	
Median (IQR)	5 (3, 6)	5 (3, 6)		5 (3, 6)
***Comorbidities/risk factors, n (%)***				
Diabetes	33 (14.0%)	16 (8.2%)	0.056	49 (11.4%)
Severe obesity (BMI>35)	33 (13.9%)	20 (10.2%)	0.240	53 (12.2%)
Obesity (BMI>30)	72 (30.4%)	46 (23.5%)	0.108	118 (27.3%)
Hypertension	97 (41.5%)	83 (42.6%)	0.817	180 (42.0%)
Cardiovascular disease	42 (17.9%)	36 (18.5%)	0.891	78 (18.2%)
Cerebrovascular disease	10 (4.3%)	10 (5.1%)	0.669	20 (4.7%)
Chronic respiratory disease	37 (15.7%)	34 (17.3%)	0.642	71 (16.4%)
Renal impairment	4 (1.7%)	1 (0.5%)	0.250	5 (1.2%)
Neurologic disease	7 (3.0%)	9 (4.6%)	0.378	16 (3.7%)
Autoimmune disease	24 (10.2%)	21 (10.7%)	0.865	45 (10.4%)
Neoplasms	19 (8.1%)	15 (7.7%)	0.879	34 (7.9%)
Hematologic disease	13 (5.5%)	13 (6.7%)	0.623	26 (6.0%)
Immunodeficiency	11 (4.9%)	9 (4.7%)	0.924	20 (4.8%)
***Vital signs at baseline***				
SpO2, median (IQR)	97 (96, 98)	97 (96, 98)	0.289	97 (96, 98)
Fever (>37.5°C), n(%)	12 (5.1%)	14 (7.3%)	0.342	26 (6.1%)
BMI, median (IQR)	26.67 (23.71, 31.89)	25.92 (23.10, 30.12)	0.079	26.23 (23.46, 31.22)
***Laboratory values, median (IQR)***				
Ferritin, ng/ml	161.5 (68.00, 274.0)	179.0 (110.0, 313.0)		173.0 (81.00, 296.0)
C-reactive protein, mg/dl	1.33 (0.52, 3.19)	1.20 (0.49, 2.42)		1.27 (0.50, 2.83)
Lymphocytes,/uL	1210 (850.0, 1600)	1160 (880.0, 1530)		1180 (870.0, 1560)
***Baseline SARS-COV-2***				
***Serology, n (%)***			<.001	
Positive	104 (43.9%)	112 (57.1%)		216 (49.9%)
Negative	74 (31.2%)	80 (40.8%)		154 (35.6%)
Unknown	59 (24.9%)	4 (2.0%)		63 (14.5%)
***Vaccination, n (%)***			0.077	
Yes (partly or fully)	140 (60.6%)	101 (52.1%)		241 (56.7%)
***Vaccine type, n (%)***			0.050	
BNT162b2	75 (68.8%)	65 (68.4%)		140 (68.6%)
mRNA-1273	18 (16.5%)	6 (6.3%)		24 (11.8%)
ChAdOx1	8 (7.3%)	20 (21.1%)		28 (13.7%)
Ad26.COV2.S	8 (7.3%)	4 (4.2%)		12 (5.9%)
Other/unknown	31 (22.1%)	6 (5.9%)		37 (15.4%)
***SARS-COV-2 variant, n (%)***			0.886	
B.1.1.7/Alpha	34 (22.5%)	37 (21.4%)		71 (21.9%)
P.1/Gamma	14 (9.3%)	11 (6.4%)		25 (7.7%)
B.1617.2/Delta	87 (57.6%)	105 (60.7%)		192 (59.3%)
Other VoC	1 (0.7%)	1 (0.6%)		2 (0.6%)
Not done	15 (9.9%)	19 (11.0%)		34 (10.5%)
***Baseline CT, mean (SD)***	21.01 ± 6.46	20.14 ± 6.09		20.56 ± 6.28
***MASS score, median (IQR)***	2 (0, 4)	2 (0, 3)	0.104	2 (0, 4)
***Enrolled after June 2021, n (%)***	172 (72.6%)	127 (64.8%)	0.082	299 (69.1%)

^*^Chi-square or Mann-Whitney test as appropriate.

BAM/ETE, bamlanivimab/etesevimab; CAS/IMD, casirivimab/imdevimab; IQR, interquartile range; BMI, body mass index; SpO2, peripheral oxygen saturation; MASS, Monoclonal Antibodies Screening Score.

### Primary Endpoint

COVID19-related hospitalization or death from any cause occurred in 19 participants: 15 patients in the BAM/ETE group (6.3%) and 4 patients in CAS/IMD group (2%) (**Table 2**). Two deaths were observed, both in patients treated with BAM/ETE experiencing COVI-19 clinical progression.

**Table 2:** Main outcomes by intervention groups.

A. Main outcomes by intervention group				
Outcomes	Regimen started			
	BAM/ETE	CAS/IMD	p-value^*^	Total
	N= 237	N= 196		N= 433
***Primary Outcome*** ^a^ ***, n(%)***	15 (6.3%)	4 (2.0%)	0.030	19 (4.4%)
***^1st^ Secondary Outcome*** ^b^ ***, n(%)***	21 (8.9%)	8 (4.1%)	0.048	29 (6.7%)
***^2nd^ Secondary Outcome*** ^c^ ***, n(%)***	9 (3.8%)	2 (1.0%)	0.068	11 (2.5%)

BAM/ETE, bamlanivimab/etesevimab; CAS/IMD, casirivimab/imdevimab.

a Patients with COVID-19 related hospitalization or death for any cause over days 0-30.

b Patients with hospitalization or death for any cause over days 0-30.

c Patients with hospitalization or death for any cause over days 3-30.

^*^Chi-square.

**Figure 3:**
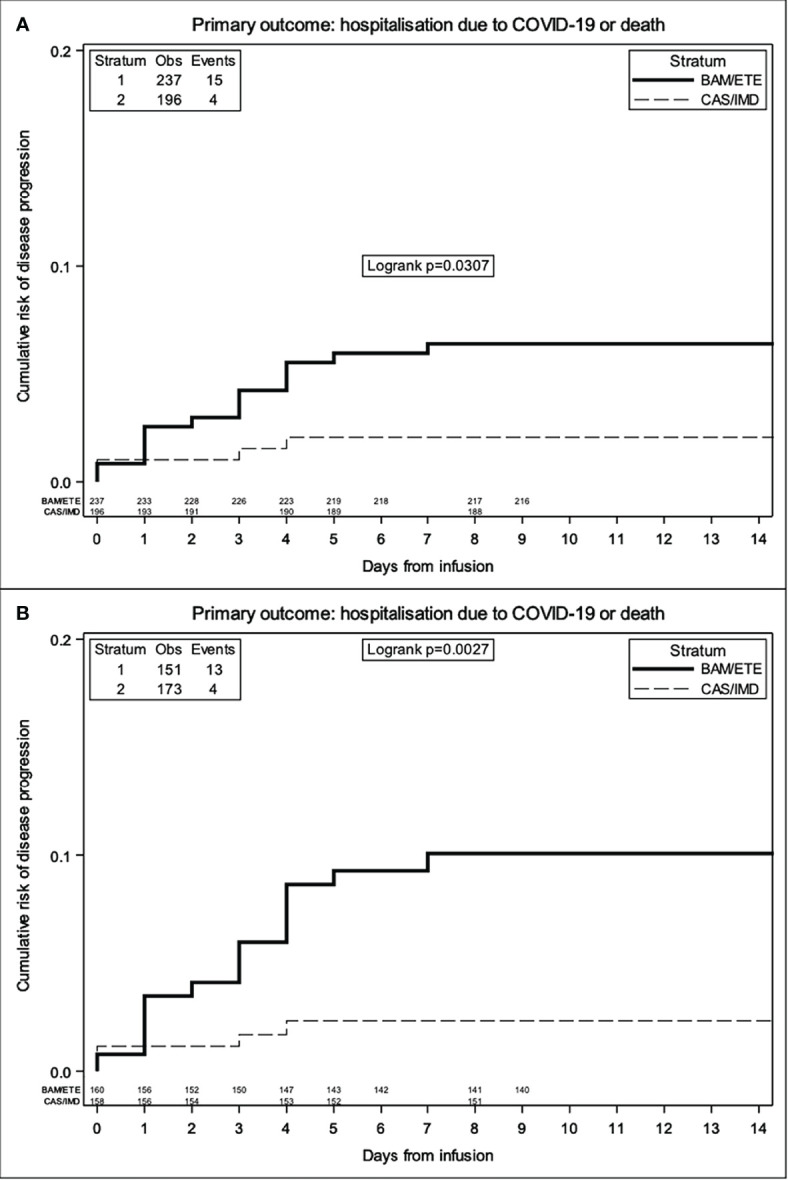
Unweighted **(A)** and Weighted **(B)** Kaplan Meier curves of time to primary endpoint by intervention group -weights include month of enrolment and type of VoC.

The majority of the events occurred before D7. In the weighted Kaplan-Meier analysis there was greater evidence for a difference in risk by intervention group (>10% for BAM/ETE) than in the unweighted (**Figures 3A, B**).

The relative hazard of COVID-19-related hospitalization or death for CAS/IMD compared to BAM/ETE was 0.28 (95%CI 0.09-0.85; p=0.024, **Table 3A**).

**Table 3:** Relative hazard from fitting a marginal model using BAM/ETE as the comparator group for primary (A) and secondary endpoints (B, C).

A. RH from fitting a marginal model for primary endpoint												
			Unadjusted and adjusted marginal relative hazards of hospitalization/death									
			Unadjusted HR (95% CI)			p-value			Adjusted^**^ HR (95% CI)			p-value
			**Primary endpoint** ^a^									
BAM/ETE			1.00						1.00			
CAS/IMD			0.32 (0.10, 0.95)			0.041			0.28 (0.09, 0.85)			0.024
**B. RH from fitting a marginal model for 1st secondary endpoint**												
			**1st secondary endpoint** ^b^										
BAM/ETE			1.00						1.00				
CAS/IMD			0.45 (0.20, 1.02)			0.056			0.40 (0.17, 0.91)			0.029
**C. RH from fitting a marginal model for 2nd secondary endpoint**												
			**2nd Secondary endpoint** ^c^											
BAM/ETE			1.00						1.00					
CAS/IMD			0.26 (0.06, 1.22)			0.088			0.22 (0.05, 1.00)			0.051

BAM/ETE, bamlanivimab/etesevimab; CAS/IMD, casirivimab/imdevimab.

a Patients with COVID-19 related hospitalization or death for any cause over days 0-30.

b Patients with hospitalization or death for any cause over days 0-30.

c Patients with hospitalization or death for any cause over days 3-30.

^**^adjusted for calendar month, MASS score and VoCs.

### Secondary Endpoints

Hospitalization or death from any cause by day 30 occurred in 29 participants (9% in BAM/ETE vs. 4% in CAS/IMD). Overall, 18 of these secondary events (62%) occurred within day three after infusion (**Table 2**).

**Tables 3B,C**, showed the weighted relative hazard ratio for secondary endpoints.

### Effect Measure Modification Analysis

**Figure 4:**
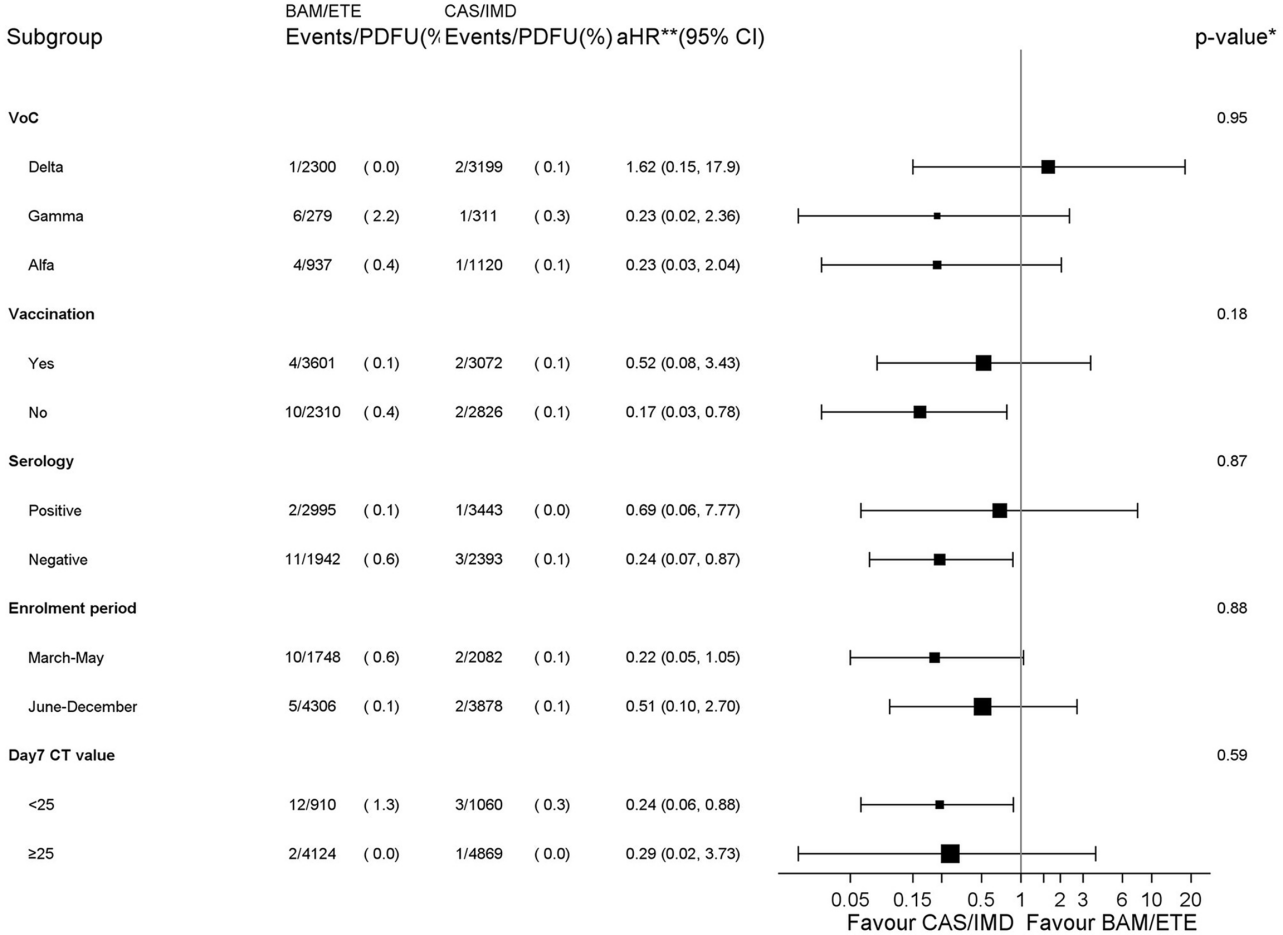
Forest plot of the effect of the intervention by subsets - Primary endpoint. *p-value corresponds to the test for interaction between intervention and each subgroup unadjusted for multiplicity. **aHR1– (vaccination, serology and CT strata) adjusted for calendar month and VoCs; **aHR2 – (VoCs stratum) adjusted for MASS score because of a positivity issue leading to abnormally high weights in the stratum of participants infected with the Delta VoC; **aHR3 – (enrolment period stratum) adjusted for MASS score and VoCs. PDFU, person days of follow-up.

The analysis by subsets carried no evidence that the effect of the intervention was different across a number of stratification variables for the primary endpoint (p-values for interaction >0.18, **Figure 4**). The effect of the intervention appeared attenuated in participants with the Delta VoC but with large uncertainty around these subgroup estimates.

### Analysis of Covariance

**Figure 5:**
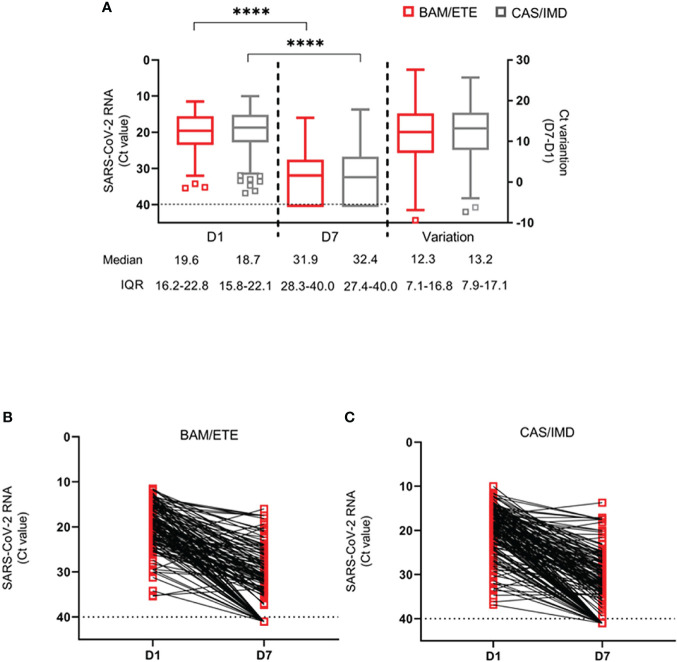
SARS-CoV-2 RNA levels at D1 and D7 in patients treated with CAS/IMD and BAM/ETE. **(A)** Box-and-whiskers plot showing the comparison of viral loads detected at D1 and D7 in patients treated with BAM/ETE (n=170 and 154, respectively) or CAS/IMD (n=183 and 174, respectively), and the variation of RNA levels observed between the two time-points by intervention (BAM/ETE, n=145; CAS/IMD, n=165). Viral RNA levels are expressed as CT of Orf1ab gene amplification. Median CT values and IQR are shown. Statistical analysis of the comparisons between treatment groups was performed by Mann–Whitney test: D1, p=0.29; D7, p=0.87; and Variation, p=0.71. CT values at D1 and D7 within each group were compared using paired Wilcoxon sign-rank test, ****p<0.0001. **(B, C)** Spaghetti-plot showing the Orf1ab CT values measured at D1 and D7 in each patient treated with BAM/ETE (n= 145, B) or CAS/IMD (n=165, **C**). Horizontal dashed line represents the limit of detection (CT: 40.0), values ≥40 are considered negative.

Median (IQR) of CT at D1 and D7 and CT increase between D1 and D7 are shown in **Figures 5A, B**. We found no evidence for a difference in D7 CT mean values comparing CAS/IMD vs. BAM/ETE (+0.17, 95%: -1.41; +1.74, p=0.83). Of note, proportions of participants with a negative SARS-COV-2 RT-PCR in NPS and with a high viral load at D7 were 26.1% in BAM/ETE vs. 25.8% in CAS/IMD (p=0.94) and 22.1% in BAM/ETE vs. 18.9% in CAS/IMD (p=0.46), respectively. Among patients who experienced primary outcome, none showed a negative SARS-COV-2 RT-PCR in NPS (p=0.009) and 82.4% showed still high viral load (p<0.001) on D7.

### Adverse Events

Thirty-one participants reported adverse events; a breakdown of the most common adverse events reported is described in **Table 4**. Overall, 19 events (10%) in the CAS/IMD group and 6 events (3%) in the BAM/ETE group were considered related to mAb infusion (p=0.001). One patient only (0.2%) reported severe dyspnea requiring hospitalization: this event was not considered by the investigators to be related to treatment.

**Table 4:** Proportions of participants reporting specific adverse events by intervention group.

Characteristics	Regimen started			
	BAM/ETE	CAS/IMD	p-value^*^	Total
	N= 237	N= 196		N= 433
***Adverse events, n(%)***			0.064	
Mild	8 (3.4%)	15 (7.7%)		23 (5.3%)
Moderate	0 (0.0%)	7 (3.6%)		7 (1.6%)
Severe	1 (0.4%)	0 (0.0%)		1 (0.2%)
Drug related	6 (2.5%)	19 (9.7%)	0.001	25 (5.8%)
***Individual events, n(%)***				
Arthomyalgia	1 (0.4%)	2 (1.0%)	0.455	3 (0.7%)
Asthenia	0 (0.0%)	0 (0.0%)		0 (0.0%)
Chills	0 (0.0%)	3 (1.5%)	0.056	3 (0.7%)
Diarrhea	1 (0.4%)	1 (0.5%)	0.893	2 (0.5%)
Fever	4 (1.7%)	17 (8.7%)	<.001	21 (4.8%)
Nausea	0 (0.0%)	4 (2.0%)	0.027	4 (0.9%)
Sight decrease	0 (0.0%)	1 (0.5%)	0.271	1 (0.2%)
Headache	0 (0.0%)	1 (0.5%)	0.271	1 (0.2%)
Dyspnea	1 (11.1%)	1 (4.5%)	0.506	2 (6.5%)
Abdominal pain	1 (11.1%)	0 (0.0%)	0.118	1 (3.2%)
Hematoma	0 (0.0%)	0 (0.0%)		0 (0.0%)
Paraesthesia	1 (0.4%)	0 (0.0%)	0.363	1 (0.2%)
Itch	0 (0.0%)	0 (0.0%)		0 (0.0%)
Skin rash	0 (0.0%)	2 (1.0%)	0.119	2 (0.5%)
Dizziness	0 (0.0%)	0 (0.0%)		0 (0.0%)
Vomit	0 (0.0%)	1 (0.5%)	0.271	1 (0.2%)

^*^Chi-square or Mann-Whitney test as appropriate.

BAM/ETE, bamlanivimab/etesevimab; CAS/IMD, casirivimab/imdevimab; IQR, interquartile range; BMI, body mass index; SpO2, peripheral oxygen saturation.

## Discussion

To our knowledge, this analysis is the first real-life comparison of two routinely used anti-SARS-CoV-2 mAbs by a trial emulation methodology using observational data collected from outpatients with early mild-to-moderate COVID-19 and at high-risk for disease progression. In an evolving scenario of SARS-CoV-2 variants from Alpha to Delta, we found that patients receiving Casirivimab/Imdevimab had a 72% lower risk of COVID-19 related-hospitalization or death from any cause than patients receiving Bamlanivimab/Etesevimab. The greater benefit of Casirivimab/Imdevimab was evident also excluding patients who experienced failure by day2 (a time window in which events cannot be ascribed to lack of treatment effect), with a confirmed 78% risk reduction.

Overall, we found no evidence that the magnitude of the difference of the effect between mAb interventions on the risk of clinical outcomes was different in specific subsets of the study population. Nevertheless, the effect of Casirivimab/Imdevimab appeared to be larger in subgroups with the highest known risk of disease progression: patients with higher baseline viral load (23), unvaccinated (24, 25) and those enrolled before June 2021, when the target population included people with higher risk of severe outcome (26).

On the other hand, there was larger uncertainty around the hazard ratio comparing interventions in the subgroup of patients infected with the Delta VoC with a 95% CI not excluding superiority of Bamlanivimab/Etesevimab vs. Casirivimab/Imdevimab. This finding is consistent with *in vitro* observed retained activity of Bamlanivimab/Etesevimab (15–27) against Delta variant (B.1617.2, non-AY.1/AY.2), and supports the recommended use of both Bamlanivimab/Etesevimab and Casirivimab/Imdevimab in settings of elevated Delta VoC prevalence.

To our knowledge, this is the first analysis from real-life data evaluating virological response to mAb treatment. It has been suggested that mAbs may act as antiviral neutralizing agents through multiple mechanisms, such as targeting free virus and virally infected cells (28). Significant decrease in viral load was described in RCT (2–4) for the two mAbs combinations analyzed in this study, but relation between virological and clinical outcomes remains uncertain (29). Interestingly, our analysis showed no difference in terms of viral load reduction from D1 to D7 between Casirivimab/Imdevimab and Bamlanivimab/Etesevimab, but also displayed a very strong association between clinical and virological outcomes, suggesting that patients developing severe disease also failed in viral clearing. However, viral load was measured after the occurrence of all clinical events, so it is difficult to determine how much clinical outcome was mediated by the virological response or whether lack of virological clearance was actually a consequence of the clinical picture.

Finally, our data confirmed safety and tolerability of these two mAb combinations in a real-life unselected population.

Our analysis has some limitations. First, due to the observational nature of the study conducted in a single COVID health care center and to the lack of a randomized design, confounding bias cannot be ruled out. Further, eligibility criteria changed over time concurrently with the advent of Delta wave and with a wider use of Bamlanivimab/Etesevimab, due to available supplies. However, results were similar after controlling for MASS score in the regression models. Moreover, the lack of an early measure of CT (e.g. at D3) prevented us from investigating viral load as a potential mediator. Finally, the study was conducted before the emergence of Omicron B.1.1.529 VoC, which is going to subvert previous assessment about mAbs treatment as several *in vitro* studies suggest that both Bamlanivimab/Etesevimab and Casirivimab/Imdevimab did not retain a remarkable activity against Omicron (16–32). Despite this, even today a proportion of illnesses and consequent hospitalizations are still due to the VoCs different from Omicron, and so knowledge of comparative data between available mAbs is still crucial for optimizing treatment in pandemic times.

## Data Availability Statement

The raw data supporting the conclusions of this article will be made available by the authors, without undue reservation.

## Ethics Statement

The studies involving human participants were reviewed and approved by National Ethics Committee. The patients/participants provided their written informed consent to participate in this study.

## Author Contributions

AA, VM conceptualized and designed the study. VM and IM wrote the protocol. VM, FC and AC wrote the first draft of the manuscript and referred to appropriate literature. AC was also the main responsible person for formal data analysis. AA, AC, VM, CA, FC, CCa and SL conceived, supervised the study and contributed to data interpretation. CCi and PP were responsible for data curation. AV, IM, AG, SC, EG, EN, FV revised the manuscript content, reviewed and edited the manuscript. LE, CF, EL, FC, CCa and AG performed all virological test. IM, AV, SL and SR enrolled participants. All authors agreed with and approved the final version of the manuscript.

## Funding

The study was performed in the framework of the SARS-CoV-2 surveillance and response program implemented by the Lazio Region Health Authority. This study was supported by funds to the Istituto Nazionale per le Malattie Infettive Lazzaro Spallanzani IRCCS, Rome (Italy), from Ministero della Salute (Ricerca Finalizzata COVID-2020-12371675 and Ricerca Corrente linea 1 on emerging and reemerging infections”).

## Conflict of Interest

The authors declare that the research was conducted in the absence of any commercial or financial relationships that could be construed as a potential conflict of interest.

## Publisher’s Note

All claims expressed in this article are solely those of the authors and do not necessarily represent those of their affiliated organizations, or those of the publisher, the editors and the reviewers. Any product that may be evaluated in this article, or claim that may be made by its manufacturer, is not guaranteed or endorsed by the publisher.
